# Effect of haemodiafiltration vs conventional haemodialysis on growth and cardiovascular outcomes in children – the HDF, heart and height (3H) study

**DOI:** 10.1186/s12882-018-0998-y

**Published:** 2018-08-10

**Authors:** Rukshana Shroff, Aysun Bayazit, Constantinos J. Stefanidis, Varvara Askiti, Karolis Azukaitis, Nur Canpolat, Ayse Agbas, Ali Anarat, Bilal Aoun, Sevcan Bakkaloglu, Devina Bhowruth, Dagmara Borzych-Dużałka, Ipek Kaplan Bulut, Rainer Büscher, Claire Dempster, Ali Duzova, Sandra Habbig, Wesley Hayes, Shivram Hegde, Saoussen Krid, Christoph Licht, Mieczyslaw Litwin, Mark Mayes, Sevgi Mir, Rose Nemec, Lukasz Obrycki, Fabio Paglialonga, Stefano Picca, Bruno Ranchin, Charlotte Samaille, Mohan Shenoy, Manish Sinha, Colette Smith, Brankica Spasojevic, Enrico Vidal, Karel Vondrák, Alev Yilmaz, Ariane Zaloszyc, Michel Fischbach, Franz Schaefer, Claus Peter Schmitt

**Affiliations:** 1 Great Ormond Street Hospital for Children NHS Foundation Trust, London, WC1N 3JH UK; 2 Cukurova University, Adana, Turkey; 3 A & P Kyriakou Children’s Hospital, Athens, Greece; 4 Clinic of Paediatrics, Vilnius University, Vilnius, Lithuania; 5 Cerrahpasa School of Medicine, Istanbul, Turkey; 6 Armand Trousseau Hospital, Paris, France; 7 Gazi University Hospital, Ankara, Turkey; 8 Medical University of Gdansk, Gdansk, Poland; 9 Ege University Faculty of Medicine, Izmir, Turkey; 10 University Children Hospital Essen, Essen, Germany; 11 Hacettepe University, Ankara, Turkey; 12 University Hospital Cologne, Cologne, Germany; 13 University Hospital of Wales, Cardiff, UK; 14 Hôpital Necker-Enfants Malades, Paris, France; 15 Hospital for Sick Children, Toronto, Canada; 16 Children’s Memorial Health Institute, Warsaw, Poland; 17 Fondazione IRCCS Ca’ Granda Ospedale Maggiore Policlinico, Milan, Italy; 18 Bambino Gesù’ Children Research Hospital, IRCCS, Rome, Italy; 19 Hôpital Femme Mère Enfant, Hospices Civils de Lyon, Bron, France; 20 Hôpital Jeanne De Flandre, Lille Cedex, France; 21 Royal Manchester Children’s Hospital, Manchester, UK; 22 Evelina Children’s Hospital, London, UK; 23 Institute for Global Health, UCL, London, UK; 24 University Children’s Hospital, Belgrade, Serbia; 25 Pediatric Dialysis and Transplant Unit, Padova, Italy; 26 University Hospital Motol, Prague, Czech Republic; 27 Istanbul University Faculty of Medical, Istanbul, Turkey; 28 Children’s Dialysis Center, Strasbourg, France; 29 Center for Pediatrics and Adolescent Medicine, Heidelberg, Germany

**Keywords:** Haemodialysis (HD), Haemodiafiltration (HDF), Children, Cardiovascular, Growth, Carotid intima media thickness (IMT)

## Abstract

**Background:**

Cardiovascular disease is prevalent in children on dialysis and accounts for almost 30% of all deaths. Randomised trials in adults suggest that haemodiafiltration (HDF) with high convection volumes is associated with reduced cardiovascular mortality compared to high-flux haemodialysis (HD); however paediatric data are scarce. We designed the haemodiafiltration, heart and height (3H) study to test the hypothesis that children on HDF have an improved cardiovascular risk profile, growth and nutritional status and quality of life, compared to those on conventional HD.

We performed a non-randomised parallel-arm intervention study within the International Paediatric Haemodialysis Network Registry comparing children on HDF and conventional HD to determine annualised change in cardiovascular end-points and growth. Here we present the 3H study design and baseline characteristics of the study population.

**Methods:**

190 children were screened and 177 (106 on HD and 71 on HDF) recruited from 28 centres in 10 countries. There was no difference in age, underlying diagnosis, comorbidities, previous dialysis therapy, dialysis vintage, residual renal function, type of vascular access or blood flow between HD and HDF groups. High flux dialysers were used in 63% of HD patients and ultra-pure water was available in 52%. HDF patients achieved a median convection volume of 13.3 L/m^2^; this was associated with the blood flow rate only ((*p* = 0.0004, *r* = 0.42) and independent of access type (*p* = 0.38).

**Discussion:**

This is the largest study on dialysis outcomes in children that involves deep phenotyping across a wide range of cardiovascular, anthropometric, nutritional and health-related quality of life measures, to test the hypothesis that HDF leads to improved cardiovascular and growth outcomes compared to conventional HD.

**Trial registration:**

ClinicalTrials.gov: NCT02063776. The trial was prospectively registered on the 14 Feb 2014.

## Background

Cardiovascular disease is the most common cause of mortality in children and young adults on dialysis, accounting for 30% of deaths [1]. Chronic fluid overload and mineral dysregulation with hyperphosphataemia and hyperparathyroidism, are widely prevalent in dialysis patients. While cardiovascular morbidity begins in pre-dialysis chronic kidney disease (CKD), it is accelerated on dialysis [2, 3], as mineral bone disease, fluid overload and factors specific to the dialysis milieu are accentuated with longer dialysis vintage [4, 5]. Even within a short period of 3 months on conventional haemodialysis (HD), biomarkers of inflammation, oxidative stress and endothelial dysfunction were shown to increase [6].

Outcomes on HD cannot be further improved by increasing the flux or efficiency of dialysis [7]. Haemodiafiltration (HDF) is a newer technique of dialysis that utilises a combination of diffusive and convective solute transport through a highly permeable membrane [8–10], thereby achieving clearance of middle and large molecular weight solutes unlike conventional HD. In recent years randomised trials in adults have shown that HDF is associated with improved cardiovascular and all-cause mortality compared to HD [11–13], and a dose-response relationship has been demonstrated between the magnitude of the convection volume and survival [13]. In addition, ultra-pure dialysate that is used in HDF reduces low-grade endotoxaemia, that can develop in HD patients [14].

HDF is increasingly used in children, but there are few data on outcomes. Fischbach et al. showed improved nutrition and growth [15], reduced inflammation [16], regression of left ventricular hypertrophy [16, 17], improved anaemia control [16] and reduced post-dialysis recovery time [15] in a small number of children undergoing daily HDF. Recent studies from our group have shown that when HD patients are switched to HDF keeping all other dialysis related parameters constant, a significant improvement in inflammation, antioxidant capacity and endothelial risk profile was achieved even within a short time of 3 months on HDF [6], however longer term effect on cardiovascular outcomes is unknown. There are no prospective studies comparing the outcomes of HD with HDF in children.

We designed the haemodiafiltration, heart and height (3H) study to test the hypothesis that children on HDF have an improved cardiovascular risk profile, growth and nutritional status and quality of life, compared to those on conventional HD. The 3H study will investigate the extent and progression of cardiovascular morbidity using a broad spectrum of measures in the largest cohort of children and adolescents on dialysis assembled to date. These data will allow us to determine the optimal dialysis modality for children requiring in-centre dialysis, the modifiable risk factors for improving cardiovascular and nutritional outcomes as well as important patient related outcome measures (PROMs).

## Methods

The 3H study will systematically evaluate cardiovascular, nutritional and health-related quality of life (QoL) measures in children on HD and HDF to test the hypothesis that children on HDF have an improved cardiovascular risk profile and growth and nutritional status compared to those on conventional HD. Primary and secondary outcome measures are described in Table 1. We will assess for difference in these outcomes between the two dialysis modalities, and the impact of modifiable risk factors, including clinical and dialysis-related factors, biochemical measures and medications. PROMs will be assessed using established QoL questionnaires as well as dialysis-specific outcomes. This is a registered clinical trial (ClinicalTrials.gov: NCT02063776).

**Table 1:** Summary of data to be obtained in the 3H study

Cardiovascular measures (annual intervals)	(i) High resolution sonography of the common carotid arteries to measure intima media thickness (morphology, B-Mode) and elasticity (function, M-mode)
	(ii) Pulse wave velocity and augmentation index
	(iii) Echocardiogram
	(iv) 24-h ambulatory blood pressure monitoring
Anthropometry	
-6-monthly intervals-	(i) Weight, height, body mass index and pubertal staging
	(ii) Body composition analysis by multifrequency bioelectrical impedance analysis
Biomarker monitoring (6-monthly intervals)	
-Nutritional measures	Albumin, prealbumin, leptin, ghrelin, cholecystokinin, endogenous growth hormone production (IGF-1, IGF-binding protein), adiponectin, resting energy expenditure (calculated), normalized protein catabolic rate (calculated), physical activity index
-Cardiovascular measures	calcium, phosphate, parathyroid hormone, FGF-23 (c-terminal), soluble klotho, 25-hydroxyvitamin D, 1,25-dihydroxyvitamin D, β2 microglobulin, fetuin-A, osteoprotegerin, markers of inflammation (IL6, IL-10, high-sensitivity CRP, TNF-α, plasma intradialytic endotoxin), markers of endothelial dysfunction (homocysteine, ADMA, SDMA), markers of bone turnover (bone-specific alkaline phosphatase, sclerostin, collagen telopeptides, β-cross-laps), markers of oxidative and carbonyl stress
Quality of life questionnaires (6-monthly intervals)	(i) Paediatric Index of Emotional Distress (Pi-ED)
	(ii) Paediatric Quality of Life (PedsQL)
	(iii) Strengths Difficulties Questionnaire (SDQ)
	(iv) Patient related outcome measures related to dialysis: post-dialysis recovery time, sleep pattern, school attendance, physical activity, appetite.

### Study design

3H is a multi-centre, non-randomised parallel-arm intervention study carried out by investigators of the International Paediatric Hemodialysis Network (IPHN) Registry and the Cardiovascular Comorbidity in Childhood CKD (4C) Study [18]. The IPHN prospectively collects detailed data from children on maintenance haemodialysis via an on-line electronic system (www.pedpd.org). The infrastructure, expertise and collaborations of the 4C study consortium, that is performing longitudinal follow-up in a cohort of over 700 children with CKD, is utilised and expanded for clinical and cardiovascular monitoring in 3H.

Children were recruited from 28 paediatric dialysis units across Europe and in Toronto, Canada. A minimum follow-up of 12 months was required.

#### Inclusion criteria

1. Paediatric patients, 5–20 years of age undergoing post-dilution HDF or HD (incident and prevalent patients)
2. HD or HDF for 4 h per session 3 times per week
3. A single pool Kt/V > 1.2 in prevalent patients in the month preceding recruitment

#### Exclusion criteria

1. Children in whom a living donor kidney transplant is planned within 6 months
2. Pre-dilution HDF
3. HD or HDF of any duration other than 4 h per session 3 times per week

#### Primary end points

*1. Annualised change in carotid intima media thickness (cIMT) standard deviation score (SDS).*

*2. Annualised change in height SDS.*

#### Secondary end-points

*Secondary end-points related to cardiovascular measures, nutrition and growth, and QoL are listed in* Table 1*.*

#### HDF and HD procedures

Standardised procedures for HDF and HD were provided to all centres, but individualised changes to the dialysis prescription will be left to the treating physician. The decision to perform HD or HDF were left to the treating physicians in each centre. In order to determine the effect of convective clearance on outcome, only prevalent HD patients with single pool Kt/V > 1.2 in the preceding month were enrolled. Given that recent RCTs in adults have shown that all cause and cardiovascular mortality are lower when higher convection volumes are used [13], we will aim for a comparable target convection volume of 12-15 L/m^2^ body surface area in children. Ultrapure dialysis fluid (defined as containing < 0.1 colony-forming unit/ml (CFU/ml) and < 0.03 endotoxin unit/ml (EU/ml)) will be used as per international standards and depending on availability in each unit. HD will be performed with similar membranes using a similar blood flow rate and dialysate composition as on HDF. Pure (defined as containing < 100 CFU/ml and < 0.25 EU/ml) or ultrapure dialysate will be used. One- to 3-monthly assessments of water quality will be performed in each centre as per local protocols, and in addition, water quality at each centre will be checked in a central lab annually. The choice of dialysate sodium, bicarbonate and calcium, as well as sodium or ultrafiltration profiling, will be left to physician discretion, but noted in all cases.

### Study organization

Patients meeting the inclusion criteria will be enrolled by local investigators. Six investigators serve as regional coordinators and visit study centres annually to perform the vascular scans, collect blood samples and complete data entry. All investigators will be provided with portable equipment for imaging. To ensure optimal reproducibility and quality of the imaging, prior to the start of the study the coordinators will be trained in all the vascular imaging, and blinded assessment of the vascular measures will be performed periodically to determine intra-observer and inter-observer variability. All training of the investigators was performed as part of the 4C study [19], and 6-monthly investigator meetings will be held alongside the 4C study meetings to synchronize study activities, discuss results, and exchange experiences. In the only non-European centre, Toronto, the local radiologists will perform the vascular imaging as per study protocol, and all the scans will be analysed centrally. Standard operating procedures for IMT, PWV, ECHO and ABPM are available on the IPHN website for easy reference.

### Investigational plan

The following investigations will be performed as outlined in Table 1. All investigations will be performed before a mid-week session of dialysis and completed within a 2 week interval of each other.

#### I - cardiovascular monitoring (baseline and annual intervals)

1. cIMT - High resolution ultrasound of the common carotid artery to measure cIMT (morphology, B-Mode) and elasticity (function, M-Mode) according to the Mannheim cIMT consensus [20]. The cIMT will be obtained by five averaged measurements on each side using a portable ultrasound device (Acuson P50; Siemens Medical Solutions USA, Inc.) with integrated digital image evaluation software (Syngo US Workplace; Siemens Medical Solutions USA, Inc.). As cIMT in children changes with growth, it will be expressed as a SDS using reference values normalized for age, sex and height derived from healthy children [21].
2. Aortic pulse wave velocity (PWV) and augmentation index will be measured with the Oscillometric Vicorder device using the distance from the suprasternal notch to the femoral recording point via the umbilicus as path length. The method was validated against the gold standard of applanation tomonetry [22], and PWV SDS values normalized for age, sex and height were derived from a large European paediatric population [22].
3. Two–dimensional echocardiography (ECHO) images are obtained for the analysis of left ventricular (LV) volume, wall thickness and chamber dimensions. The LV mass will be calculated according to the Devereux Equation [23] and indexed to height (LVMI) and sex– and age–specific LVMI partition values of Khoury et al. [24] applied to define left ventricular hypertrophy (LVH). Echocardiographic assessments will be performed either by a local cardiologist or by the regional coordinator, standardized according to the guidelines of the American Society of Echocardiography [25].
4. 24-h ambulatory blood pressure monitoring (ABPM) will be performed using the Spacelab ABPM portable device (Spacelabs 90,207–2Q) as previously described [26]. The time–averaged 24-h mean arterial pressure (MAP) will be used for the analyses and hypertension defined as 24-h time–integrated MAP exceeding the 95th percentile [26]. Patients on antihypertensive medications will be referred to as having controlled or uncontrolled hypertension if their 24-h MAP is below or above the 95th percentile, respectively.
5. Bioimpedence spectroscopy will be performed using the Fresenius body composition monitoring (BCM®; Fresenius Medical Care, GmbH) device as previously described [27]. This multi-frequency bioimpedance analysis measures 50 frequencies between 5 to 1000 kHz and calculates the extracellular, intracellular and total body water. Parameters generated directly from the device namely absolute overhydration (OH, litre) and relative overhydration (Rel-OH; %) will be recorded. Using standardised definitions, overhydration is defined as Rel-OH ≥7% and severe overhydration when Rel-OH is ≥15% [28].

#### II - clinical and anthropometric data (baseline and 6-monthly intervals)

Height, weight, body mass index, waist-hip circumference and pubertal stage will be measured using standard techniques [29]. The height velocity will be calculated as the change in height over a 6-month period standardised for age.

#### III - biochemical and biomarker monitoring: (baseline and 6-monthly intervals)

Blood samples will be collected before a mid-week session of dialysis, centrifuged and stored at -80 °C in the local hospitals before shipping to a central biorepository. Analyses will be performed on batched samples in a central lab.

#### IV - health-related quality of life questionnaire (baseline and 6-monthly intervals)

Three validated information and screening questionnaires will be given to children and their parents in their local languages: the paediatric index of emotional distress (Pi-ED) screens for emotional distress (depression and anxiety), Paediatric Quality of Life (PedsQL) for health impacts on quality of life and the Strengths Difficulties Questionnaire (SDQ) for emotional well-being and pro-social relationships. In addition, information on post-dialysis recovery time, physical activity, school or college attendance and sleep pattern will be recorded by the patient. All questionnaires will be analysed by a single psychologist blinded to the clinical status of the child.

### Data acquisition and handling

Data will be collected in the IPHN Registry by on-line data entry by the local physicians assisted by the regional investigators. All patient-specific data will be pseudonymized at the local study centres and the website and database will be on a secure server containing a password-protected domain. Study performance will be reviewed annually by a data monitoring committee.

### Statistical analysis

All analyses has been decided a priori in an analysis plan. Each quantitative outcome will be assessed for normality. cIMT, PWV, 24-h MAP profiles and height will be expressed in SD scores. The co-primary endpoints of mean and standard deviation (or median/interquartile range if appropriate) annualised change between baseline and 1 year in cIMT and height SDS will be calculated, and compared between the HDF and HD cohorts using unpaired t-tests (or the non-parametric equivalent). As the data are observational, adjustment for potential confounders (country, age, gender, access type, dialysis vintage) will be made using linear regression. The adjustment for the primary analysis will be made through the construction of propensity scores, representing the likelihood of receipt of HDF (vs HD), based on the pre-specified confounders. A sensitivity analysis will be performed to ensure robustness of results, adjusting for potential confounders through a multivariable linear regression model. The annual change in secondary endpoints will be investigated similarly, but these analyses are not formally powered, and so will be considered as secondary analyses. Correlations between continuous variables will be made using Pearson correlation coefficients if variables are normally distributed, or Spearman’s rank correlation coefficients otherwise.

Both intention to treat (based on the dialysis modality HD or HDF at the start of the study) and per protocol (at least 90% of all dialysis sessions must be in the assigned group) analyses will be performed. All analyses will be performed in SAS Version 9.4 (SAS Institute Inc., Cary, NC). All statistical tests will be two sided and *p*-values of < 0.05 will be nominally considered to indicate a statistically significant difference between groups.

### Sample size

In a parallel study investigating cardiovascular disease progression in children on HD we have shown a mean (SD) increase in cIMT by 0.5 ± 0.3 SDS/year compared to 0.0 ± 0.6 SDS/year in pre-dialysis CKD patients (unpublished data). Making a conservative assumption of an average annualized change of 0.4 ± 0.5 SDS in HD vs 0.1 ± 0.5 SDS in the (presumably) more stable HDF group, 69 children per group were required at 90% power and 2.5% Type I error (resulting in a 5% overall Type I error for the two primary endpoints; Bonferroni correction). Accounting for a 10% dropout rate, 76 subjects per group are needed. No paediatric longitudinal data are available regarding changes in height SDS or functional parameters such as PWV, and so no formal power calculation can be performed. On interim analysis at 1-year a significantly higher drop-out rate (due to transplantation) was noted in the HDF compared to the HD group. Hence, with permission from the Institutional Review Board, we extended the recruitment period by a further 12 months, and 5 new centres joined the study.

#### Ethical aspects

The study was performed according to the principles of the declaration of Helsinki. The patient information and consent forms (translated into the national languages) were reviewed and approved by the local Institutional Review Boards in each participating centre. Appropriate measures were used to guarantee maximal data confidentiality. All patient-related clinical data, including vascular scans and blood samples, was pseudonymized locally. Neither the laboratories nor the central office were able to identify individual patients, and no investigator other than the local physicians at each site were able to correlate results with clinical or laboratory data or vascular imaging.

### Baseline characteristics

One hundred ninety children were recruited from 28 centres in 10 countries (Turkey 48, United Kingdom 40, France 22, Italy 20, Germany 19, Greece 16, Serbia 8, Poland 7, Canada 8 and Czech Republic 2). Thirteen children did not meet the inclusion criteria and were excluded from further analysis (age < 5 years in 1, dialysis frequency or duration not 4 h 3 times per week in 7, pre-dilution HDF in 2, ultrapure water not used for HDF in 2 and transplantation on the day of the study in 1).

Baseline characteristics of the 177 children (106 on HD and 71 on HDF) who entered the study are described in Table 2. There was a higher prevalence of girls on HDF, but no difference was seen in age, ethnicity, underlying renal disease or presence of comorbidities. The number of children with residual urine output were similar between HD and HDF groups.

**Table 2:** Demographics of the study population at baseline

	Haemodialysis n (%) or Median (IQR)	Haemodiafiltration n (%) or Median (IQR)	p
Number	106	71	
Age			
5–10	22 (20.8)	15 (21)	0.78
10–15	37 (34.9)	27 (38)	
15–20	47 (44.3)	29 (41)	
Gender (female)	45 (42)	43 (60)	0.03
Ethnicity			
Caucasian / Asian / African /other	66 / 21 / 9 /’10	46 / 17 / 5 / 3	0.42
Underlying renal diagnosis			
Dysplasia	54 (51)	32 (45)	0.76
Glomerulonephritis	25 (24)	19 (26)	
Cystic kidney disease	4 (4)	5 (7)	
Other	17 (15)	11 (16)	
Unknown	6 (6)	4 (6)	
Presence of comorbidities			0.81
Impaired cognitive development	22 (21)	14 (20)	
Impaired motor development	11 (10)	6 (8)	
Ocular or hearing abnormalities	18 (17)	11 (15)	
Other abnormalities	25 (24)	13 (18)	
Confirmed genetic disorder	17 (16)	12 (17)	
Previous dialysis	35 (33)	34 (48)	0.07
Modality (PD / HD / both)	22 / 8 / 5	14 / 14 / 6	0.17
Cumulative time on dialysis before study (months)	24 (10, 52)	28 (16, 45)	0.92
Previous transplant	18 (17)	21 (29)	0.06
Time with functioning graft (mts)	33 (16, 96)	66 (12, 114)	0.96
Residual urine volume (ml/day)			
< 200 / 200–500 / > 500	66 (62) / 22 (21) / 18 (17)	44 (62) / 13 (18) / 14(20)	0.85
Details of dialysis therapy			
Filter			
High flux / Mid flux / Low flux	67 (63) / 24 (23) / 15(14)	71 (100)	< 0.00001
Dialysis water quality^a^			
Pure vs ultrapure	51 (48) / 55 (52)	71 (100)	< 0.00001
Vascular access^b^			
AVF / CVL / AVG	34 (33) / 71 (67) / 1 (1)	27 (38) / 42 (59) / 2(3)	0.34
Blood flow /m^2^ body surface area	182 (148, 215)	174 (144, 205)	0.40
Dialysate sodium			
≤ 138 mmol/l	76 (72)	49 (69)	0.79
> 138 mmol/l	30 (28)	22 (31)	
Dialysate bicarbonate			
32–35 mMol/L	68 (64)	42 (59)	0.48
36–42 mMol/L	38 (36)	29 (41)	

IQR – interquartile range; ^a^Dialysate water quality: Pure dialysis fluid is defined as containing < 100 colony-forming unit/ml (CFU/ml) and < 0.25 endotoxin unit/ml (EU/ml). Ultrapure dialysis fluid is defined as containing < 0.1 CFU/ml and < 0.03 EU/ml; ^b^Vascular access: AVF – arteriovenous fistula; CVL – central venous line; AVG – arteriovenous graft

### Details of dialysis therapy

Dialysis related parameters in the HD and HDF groups are described in Table 2. Ultra-pure dialysate was used for all HDF patients but less consistently in HD patients, (*n* = 71, 100% vs *n* = 55, 52%; *p* < 0.0001). High-flux dialysers were used in 67 (63%) of HD patients. There was no difference in the dialysate sodium, calcium or bicarbonate concentration between groups. The distribution of vascular access types (CVL, AVF and AVG) was comparable between the groups.

**Fig. 1:**
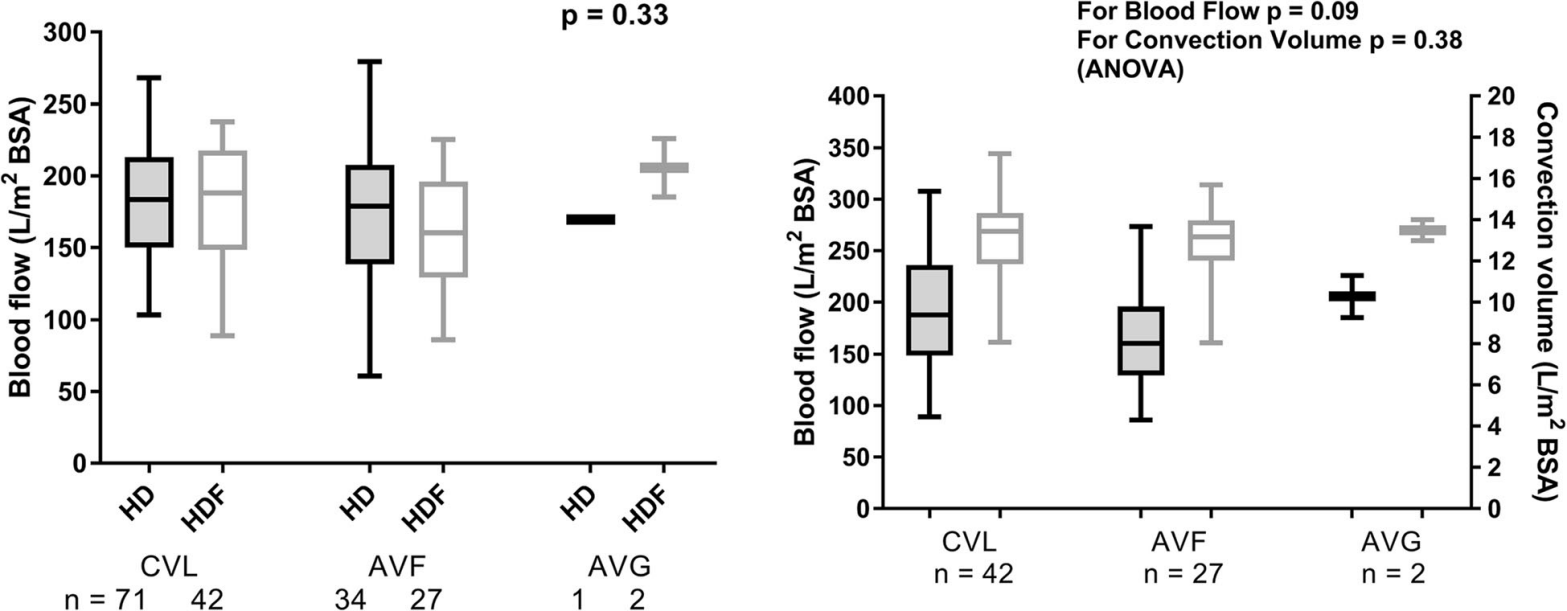
**a** Blood flow, expressed as litres/m^2^ body surface area, in children on HD and HDF with central venous lines (CVLs), arteriovenous fistulae (AVFs) and arteriovenous grafts (AVGs). **b** The relationship between blood flow and convection volume in children on HDF with CVLs, AVFs and AVGs. Convection volume is independent of the type of vascular access

**Fig. 2:**
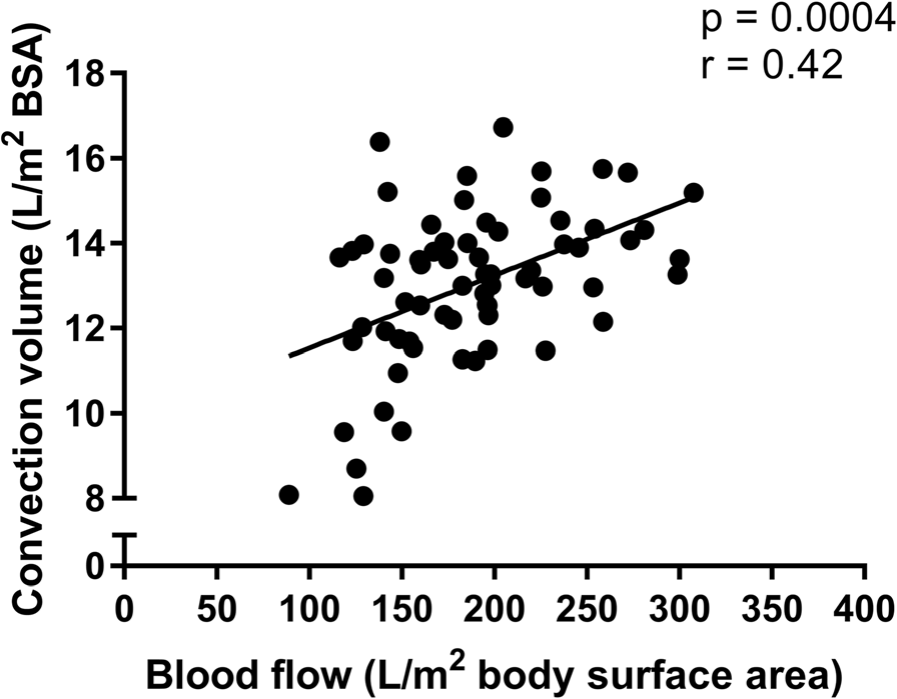
Convection volume showed a linear association with blood flow, expressed as litres/m^2^ body surface area

The median blood flow rate (standardised to body surface area) was similar between HD and HDF groups (182 [148–215] vs 174 [144–205] L/m^2^; *p* = 0.4) and was independent of the type of vascular access (*p* = 0.09; Fig. 1a). The median convection volume achieved in the HDF group was 13.3 (IQR 11.5 to 14.2) L/m^2^. The convention volume showed a strong linear relation with the blood flow rate (*p* = 0.0004, *r* = 0.42; Fig. 2), but was independent of the type of vascular access (*p* = 0.38; Fig. 1b), age or gender.

## Discussion

The 3H study is a multi-centre prospective clinical trial testing the hypothesis that HDF improves cardiovascular, nutritional and health-related QoL outcomes compared to conventional HD in children.

Conventional HD is based on diffusive transport alone and cannot remove middle-molecular-weight solutes efficiently even with the use of high-flux membranes. Middle-molecular-weight uraemic toxins include β2-microglobulin, adiponectin, leptin, ghrelin, cholecystokinin and many others hormones and inflammatory cytokines [30]. Middle-molecular-weight toxins mediate inflammation, oxidative stress, endothelial dysfunction and cardiovascular disease and have been associated with increased mortality in adults on dialysis [31]. As the importance of uraemic toxins is recognized, the need for alternative therapies that provide better removal of those solutes has become evident. HDF is a blood purification therapy combining diffusive and convective solute transport using high-flux membrane [14]. Convective transport is achieved by an effective convection volume of at least 20% of the total blood volume processed which is replaced by infusion of a sterile, non-pyrogenic solution [14].

Middle and large molecular weight compounds such as β2-microglobulins that normally accumulate on HD have > 70% better removal on HDF [32, 33]. Plasma phosphate has a 30% greater clearance by HDF [34]. The use of ‘ultrapure’ dialysate and increased removal of inflammatory cytokines [35] reduces inflammation and oxidative stress. Erythropoietin sensitivity is improved, possibly as a result of reduced inflammation and removal of erythropoiesis-inhibiting factors [34]. In HDF cooling of the patient occurs because of the large volumes of fluid that are infused, reducing the incidence of intradialytic hypotension, improving intradialytic hemodynamic stability [36], allowing faster recovery time post-dialysis, and explaining, in part, the improved cardiovascular outcomes [37].

Several prospective observational studies in adults have shown that HDF reduces cardiovascular and all-cause mortality, and recently the results of three large RCTs, collectively encompassing more than 2400 adults on dialysis, were published. Neither the CONvective TRAnsport STudy (CONTRAST) [38], nor the Turkish HDF study [12] showed a priori that there was any significant difference in all-cause mortality or cardiovascular events (both fatal and non-fatal) between HDF and HD. However, the Estudio de Supervivencia de Hemodiafiltracion On-Line (ESHOL), the RCT with the highest achieved convection volumes, reported superiority of HDF over HD with respect to all-cause and cardiovascular mortality [11]. Notably, post hoc analyses of all three studies suggested a dose-effect relationship between convection volume and mortality risk, even after adjustment for potential confounders [11, 12, 38]. Since data from the RCTs is inconsistent, a pooled individual participant data analysis of RCTs showed that HDF reduces the risk of mortality compared with conventional HD [13]. The mechanisms for improved survival are not clear, and it is not known if equivalent benefits can be expected in children on HDF.

HDF has been performed in children since the mid-70s, and is shown to be a safe and well tolerated treatment [16]. However, there are few children on HDF across the world. According to a survey of 51 paediatric dialysis units across Europe HDF is practiced only in 24 (47%) centres, with 12% of all children on extracorporeal dialysis receiving HDF [39]. In order to meet the numbers required in each arm of the study a multinational approach, involving as many centres that perform HDF as possible, was required. We utilised and extended the infrastructure developed in the ongoing 4C study [18], extending the catchment area to include other countries and centres too. However, due to small numbers we had to include both incident and prevalent dialysis patients. Randomisation was not possible as many centres were not willing to switch HDF patients back to HD, largely due to patient preference. Instead, a clinical trial within the IPHN registry was performed, and propensity matching analysis will be undertaken to adjust for centre bias. Although a rigorous study design was developed, not all dialysis centres were able to use high-flux dialysers or ultra-pure water; effects of dialysis membrane and dialysate purity will be examined by multivariable analysis. Given that children may switch between HD and HDF, both intention-to-treat and ‘as-treated’ analyses will be performed, ensuring that each patient has received at least 90% of their dialysis treatment in the assigned modality.

The convection volume is one of the key parameters of HDF prescription and outcomes as shown in the pooled study [13]. In the 3H study we were able to achieve median convection volumes of 13.4 L/m^2^ in children, which is comparable to the 23 L per 1.73 m^2^ per session that proved beneficial in the pooled study. Importantly, the convection volume was independent of patient related factors such as age, gender, access type or dialyser used, but strongly correlated with the blood flow rate, implying that convection volume is a modifiable factor that can be manipulated and optimised by the dialysis team.

As with all paediatric studies, hard end-points are fortunately rare, and surrogate measures of early cardiovascular disease have been used. Our primary end-point, change in cIMT, is a well-established surrogate measure of the extent of coronary artery disease, correlating with coronary angiography and intravascular ultrasound results [40]. It has been correlated with hard endpoints such as myocardial infarction and stroke in adults without CKD [41] and cardiovascular events in CKD [42] and dialysis [43] patients. Similarly, increased PWV has been associated with higher mortality rates even in young adults on dialysis [44].

The 3H study will also examine factors related to growth and nutrition and their association with dialysis modality, if any. Despite major advances in the understanding and therapy of uraemic growth failure, 35 to 50% of children with end-stage kidney disease still grow up to become small adults with a final height below the third percentile of the general population [45]. In a previous study in children, HDF was associated with impressive catch-up growth achieving a normal height, at or above their target mid-parental height [15]. However, this small single centre study utilised 6 days per week HDF in the pre-dilutional mode, where the replacement fluid is infused upstream of the dialyser, allowing higher filtration rates than are possible than with post-dilution HDF. Daily HDF improved appetite and corrected metabolic acidosis, but other hypothetical mechanisms for improved growth may also include reduced inflammatory cytokine release improving target tissue sensitivity to growth hormone, and superior removal of accumulated endogenous somatomedin and gonadotropin inhibitors, leading to an overall anabolic state [46]. The effect of thrice-weekly post-dilutional HDF on growth has not been studied; the 3H study will address growth outcomes, and also investigate biomarkers of nutritional and growth in HD and HDF patients.

Taken together, the 3H study is the largest prospective study in children on dialysis to date. It includes deep phenotyping across a range of cardiovascular, anthropometric, nutritional and health-related QoL measures in a quest to find modifiable risk factors that are associated with improved outcomes in dialyzed children. If HDF does improve outcomes in children, as it is shown to do in adults, dialysis physicians and commissioners would need to consider the widespread adoption of HDF therapy for children.
